# Randomized trial of physiotherapy and hypertonic saline techniques for sputum induction in asthmatic children and adolescents

**DOI:** 10.6061/clinics/2020/e1512

**Published:** 2020-01-20

**Authors:** Egberto Luiz Felicio-Júnior, Viviani Barnabé, Francine Maria de Almeida, Monise Dematte Avona, Isabella Santos de Genaro, Adriana Kurdejak, Miriam Cardoso Neves Eller, Karina Pierantozzi Vergani, Joaquim Carlos Rodrigues, Iolanda de Fátima Lopes Calvo Tibério, Milton de Arruda Martins, Beatriz Mangueira Saraiva-Romanholo

**Affiliations:** I Hospital do Servidor Publico do Estado de Sao Paulo (IAMSPE), Sao Paulo, SP, BR; II Laboratorio de Terapeutica Experimental (LIM-20), Faculdade de Medicina FMUSP, Universidade de Sao Paulo, Sao Paulo, SP, BR; III Universidade Cidade de Sao Paulo (UNICID), Sao Paulo, SP, BR; IV Departamento de Pediatria e Pneumologia, Instituto da Criança, Hospital das Clinicas HCFMUSP, Faculdade de Medicina, Universidade de Sao Paulo, Sao Paulo, SP, BR

**Keywords:** Asthma, Children, Hypertonic Saline Solution, Sputum Induction, Physical Therapy Techniques

## Abstract

**OBJECTIVES:**

This study aimed to analyze the efficiency of physiotherapy techniques in sputum induction and in the evaluation of pulmonary inflammation in asthmatic children and adolescents. Although hypertonic saline (HS) is widely used for sputum induction (SI), specific techniques and maneuvers of physiotherapy (P) may facilitate the collection of mucus in some asthmatic children and adolescents.

**METHODS:**

A randomized crossover study was performed in patients with well-controlled asthma, and 90 sputum samples were collected. Children and adolescents were assessed using spirometry and randomized at entry into one of three sputum induction techniques: (i) 3% hypertonic saline - HS technique; (ii) physiotherapy (oscillatory positive expiratory pressure, forced expiration, and acceleration of expiratory flow) - P technique; and (iii) hypertonic saline + physiotherapy - HSP technique. ClinicalTrials.gov: NCT03136042.

**RESULTS:**

The total cells (mL) and the percentage (%) of differential inflammatory cells were similar in all techniques. The sputum weight (g) in the HSP technique was significantly higher than that in the HS technique. In all techniques, the percentage of viable cells was >50%, and there was no difference between the HS and P techniques. Moreover, sputum induction did not cause any alterations in the pulmonary function of patients.

**CONCLUSION:**

The physiotherapy sputum collection technique was effective in obtaining viable cells from mucus samples and yielded the same amount of sputum as the gold standard technique (hypertonic saline). In addition, the physiotherapy maneuvers were both safe and useful for sputum induction in asthmatic children and adolescents with well-controlled asthma.

## INTRODUCTION

Asthma is a chronic respiratory disease that affects 1% to 18% of the population in different countries (1). In Brazil, asthma is one of the respiratory diseases with the highest mortality rates (five deaths per day) and is the reason for >120,000 hospitalizations per year (2).

The principal triggers of asthma include viral infections, environmental or occupational allergens, smoking, exercise, and stress (3). Asthma exacerbations are characterized by airway obstruction, inflammation, and airway hyper-responsiveness (4). Although asthma is mainly diagnosed on the basis of clinical findings, a pulmonary function test needs to be performed to confirm airway obstruction, particularly in adolescents and adults (5). Asthma control includes the evaluation of symptoms and their reduction or resolution after treatment (5). To achieve these goals, inflammation must be controlled and the disease phenotype must be identified. With respect to the evaluation of this condition, sputum induction is a perfectly feasible diagnostic test (6) and sputum cell counts reflect the underlying disease and treatment adherence (6).

After the age of 6 years, the use of hypertonic saline to induce sputum is employed for cytology analysis, and this noninvasive technique allows for the assessment of inflammatory cells such as eosinophils and neutrophils (7).

Researchers sometimes encounter difficulties in obtaining induced sputum in stable patients, and respiratory therapy can promote mucus clearance through specific techniques and maneuvers. The wide variety of techniques for sputum induction and expectoration include huffing, oscillatory positive expiratory pressure (OPEP) therapy, forced expiratory technique (FET) (8-10), and others (11). The OPEP apparatus reduces the viscoelasticity of bronchial secretions (12), and some studies have shown that physiotherapy maneuvers are safe and have a satisfactory effect on sputum induction in patients with respiratory diseases (13,14). This study aimed to compare three different techniques of sputum induction in children with asthma and to evaluate the effectiveness of collecting induced sputum through physiotherapy maneuvers.

## METHODS

This study was approved by the Institutional Ethics Committee of the Clinical Hospital of the School of Medicine of University of Sao Paulo (protocol no. 674.125) and registered in ClinicalTrials.gov (NCT03136042). Thirty-eight asthmatic children and adolescents were recruited from the Pediatric Pneumology Outpatient Clinic of the Children’s Institute of the Clinical Hospital (University of Sao Paulo) and were asked to sign an informed consent form. The children and adolescents were between the ages of 7 and 18 years and were classified as having well-controlled asthma, according to the Global Initiative for Asthma 2017 (5). Patients using inhaled steroids in combination with long-acting beta_2_-agonists were also included. Patients diagnosed with other chronic pulmonary diseases (e.g., cystic fibrosis, ciliary dyskinesia, bronchiolitis obliterans, and bronchopulmonary dysplasia), those who were unable to perform pulmonary function tests, and those with insufficient sputum samples were excluded.

The primary aim of the study was to evaluate whether physiotherapy techniques, such as OPEP therapy, huffing, and acceleration of expiratory flow, are safe and efficient for sputum induction in children and adolescents with well-controlled asthma.

The secondary aim was to evaluate whether samples collected using 3% hypertonic saline and physiotherapy techniques are of good quality according to the acceptance criteria.

### Study design

**Figure 1:**
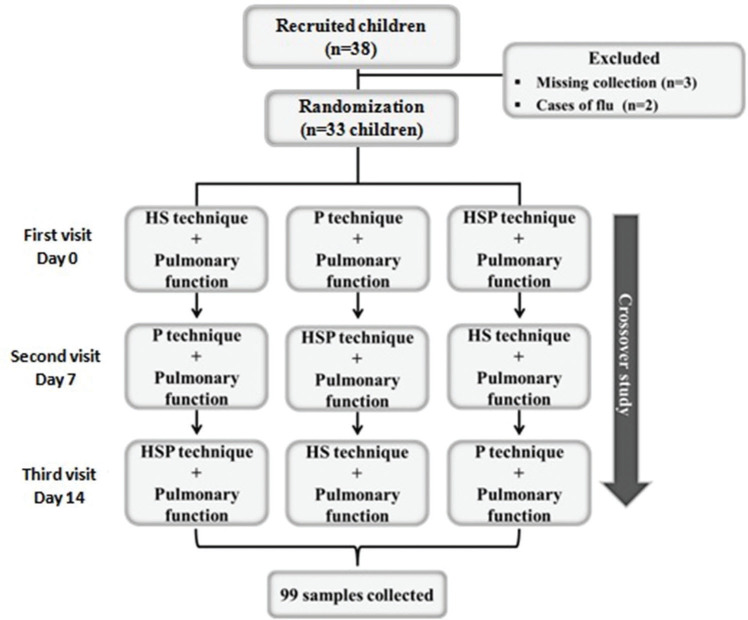
Study design. Cross-over techniques of induced sputum collection. HS: hypertonic saline; P: physiotherapy; HSP: hypertonic saline + physiotherapy.

After the participants were recruited and the informed consent form was signed, the first visit was scheduled. Thereafter, a blinded investigator randomized each participant into one of three crossover techniques as the first sputum induction protocol. The sequence of the subsequent techniques performed was as follows: (i) hypertonic saline (3%) - HS technique; (ii) physiotherapy (OPEP therapy, forced expiration, and acceleration of expiratory flow) - P technique; and (iii) hypertonic saline + physiotherapy - HSP technique. Each patient made three visits for sputum induction using each technique, with 7-day intervals to avoid interference with the results obtained with each induction (Figure 1).

Thirty-three asthmatic children and adolescents of both sexes participated in the study, and 99 samples of induced sputum were collected (Figure 1). Three children were excluded because the collected sputum samples were <2.0 mL, which was considered inadequate according to the criteria for sample acceptance (15).

### Pulmonary function

Spirometry was performed before and after the inhalation of 200 µg salbutamol (bronchodilator), in accordance with the American Thoracic Society guidelines (16), using a KoKo spirometer (PDS Instrumentation Inc., Louisville, KY, USA). If the patients did not experience respiratory discomfort or did not have a >20% decrease in pulmonary function, sputum induction was performed with one of the techniques and spirometry was repeated.

The values of forced expiratory volume in the first second (FEV_1_), peak expiratory flow (PEF), and forced vital capacity (FVC) expelled in the first second of a forced expiration (FEV_1_/FVC) were evaluated before and after bronchodilator administration and after performing the sputum induction techniques (17).

### Sputum induction

After spirometry, we collected induced sputum samples using the three techniques in all children and adolescents, as detailed below.

**Figure 2:**
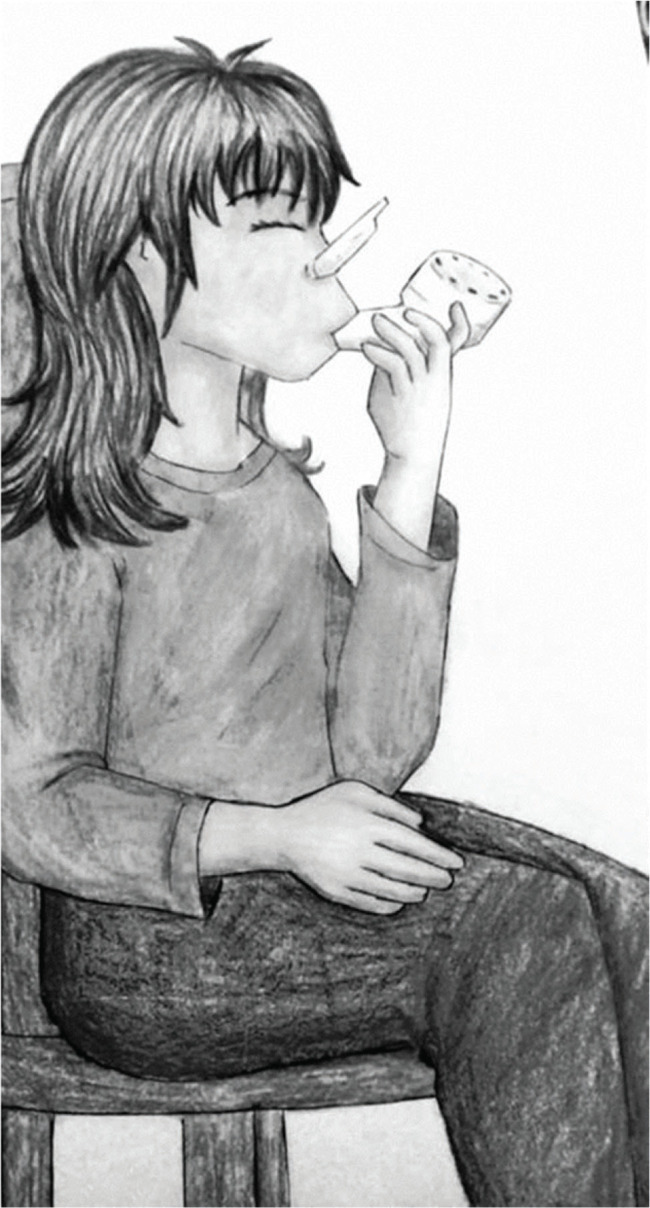
Illustrative image showing a patient performing oscillating positive expiratory pressure therapy for 5 min in a sitting position.

**Figure 3:**
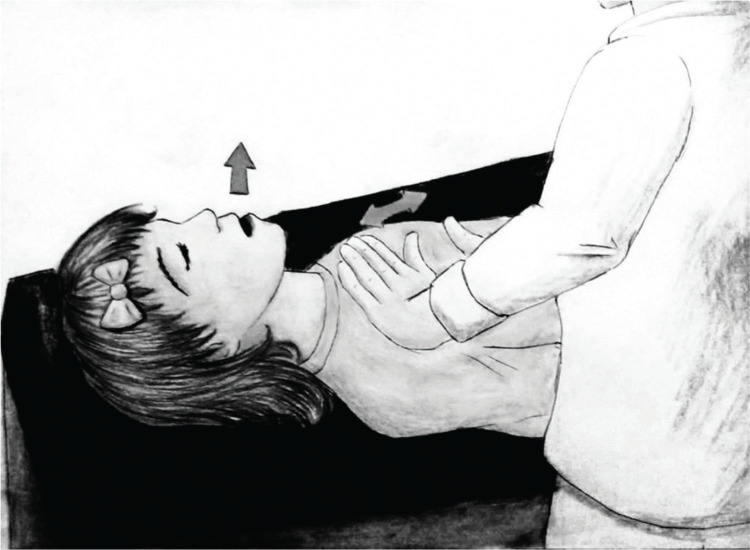
Illustrative image demonstrating the forced expiration (huffing) technique associated with accelerated expiratory flow. The therapist places one hand on the patient’s manubrium and the other hand on the xiphoid process of the patient’s sternum. Thereafter, the therapist brings the two hands together during the patient’s exhalation, accelerating the outflow of air, while the patient simultaneously exhales with the glottis and mouth open, and the abdominal muscles contracted.

(i) Sputum induction with 3% hypertonic saline (HS technique): Children and adolescents received a nebulization with hypertonic saline at a concentration of 3% for 7 min. The aerosol was generated by an ultrasonic nebulizer (Ultraneb 99; DeVilbiss, Somerset, PA, USA) with an output of 2.4 mL/min and a mass median aerodynamic diameter of 4.5 µm. If the sputum sample volume was insufficient, the nebulization could be repeated three times. After each nebulization, pulmonary function was monitored by obtaining the PEF values using the Koko spirometer. If hypertonic saline did not induce a PEF decrease (≥20%), the next nebulization was performed (17,18).

(ii) Sputum induction through physiotherapy maneuvers (P technique): The patients’ thorax was inclined by 45° in a seated position. The patients were instructed to perform calm and long exhalations through repeated OPEP therapy with a Flutter^®^ device (Scandipharm, Birmingham, AL, USA), continuously for a duration of 5 min. Thereafter, the children and adolescents were placed in a supine 0° position (Figure 2) and underwent another 5 min of FET with an open mouth/glottis (FET/huffing) in conjunction with acceleration of expiratory flow. This technique was applied by a single physiotherapist, who positioned one hand on the patient’s xiphoid process and placed the other hand on the manubrium of the patient’s sternum (Figure 3) (9). At the end of the maneuvers, all patients were advised to effectively cough into a proper sterile container.

(iii) Sputum induction with 3% hypertonic saline associated with physiotherapy maneuvers (HSP technique): First, children and adolescents underwent sputum induction through one inhalation of 3% hypertonic saline for 7 min. Second, they underwent 5 min of OPEP therapy and 5 min of huffing/FET, as described above. Third, they were advised to effectively cough into a suitable sterile container.

### Sputum processing

All samples were processed according to the previously published protocol (18). Briefly, sputum was separated from saliva and placed into a Petri dish and weighed. The samples were treated with 0.1% dithiothreitol (Sigma-Aldrich, Sao Paulo, Brazil), gently vortexed, and placed in a shaking water bath at 37°C for 20 min to ensure adequate homogenization. This suspension was filtered through a 48-µm nylon gauze (BBSH Thompson, Scarborough, Ontario, Canada) to remove cell debris and remaining mucus. The resulting clear suspension of sputum was centrifuged at 790 × g for 10 min, and the supernatant was aspirated. The total cell count was determined using a hemocytometer (Neubauer chamber). Thereafter, cytospins were prepared using a Shandom III centrifuge (Shandom Southern Instruments, Sewickley, PA, USA). Differential cell counts of 300 cells per slide were obtained after Diff-Quick staining. A blinded researcher managed all measurements, and the criteria for acceptance of the sample were inhalation time between 5 and 20 min, sputum volume ≥2 mL, sample processing time <2h, squamous cells ≤80%, and at least 200 inflammatory cells per technique (15).

### Statistical analysis

Data were analyzed using repeated-measures analysis of variance, followed by a Tukey test (post hoc test). A *p*-value of <0.05 was considered significant. Data are presented as medians and interquartile ranges. Statistical analysis was performed with SigmaPlot version 12.0 software (Armonk, NY, USA).

## RESULTS

Thirty-three asthmatic children and adolescents of both sexes (18 boys and 15 girls) participated in the study from January 2016 to July 2018. The average height was 1.44±0.18 m, and the average weight was 43.28±18.38 kg. Pulmonary function was evaluated both before and after salbutamol inhalation and after sputum induction. FEV_1_, PEF, and FEV_1_/FVC did not show any difference among the techniques (*p*≥0.05) (Table 1). None of the 33 asthmatic patients had any complications during or after the pulmonary function tests.

All sputum samples obtained using the P and HSP techniques were sufficient. In the HS technique, three children had an inadequate sputum volume and were excluded from the study in the first visit. During sputum induction with hypertonic saline, it was necessary to repeat the nebulization two times in 9 cases and three times in 10 cases; however, in most cases, only one nebulization was necessary.

The sputum induction time (Table 2) was higher with the HSP technique than with the other techniques (*p*=0.001). In all sputum samples, the percentage of viable cells was ≥80% (Table 2) and the HS technique presented higher values than the HSP technique (*p*=0.008). The sputum weight (Table 2) was higher with the HSP technique than with the HS technique (*p*=0.02). The total cell count and the percentage of differential cells (macrophages, neutrophils, lymphocytes, and eosinophils) were assessed, and the same inflammatory profile was observed among all three techniques (Table 2).

## DISCUSSION

This study compared the performance of three sputum induction methods: physiotherapy maneuvers (P technique), hypertonic saline (HS technique), and physiotherapy maneuvers plus hypertonic saline (HSP technique). Thirty-three children and adolescents participated in the study. In the HS technique, three children presented with an inadequate sputum volume and were excluded in the first visit. All sputum samples obtained using the P and HSP techniques were adequate and demonstrated good quality in accordance with the acceptance criteria. Pulmonary function was evaluated both before and after salbutamol inhalation and after sputum induction. No differences among the techniques were observed when FEV_1_, PEF, and FEV_1_/FVC were analyzed, with an increase in FEV_1_ after bronchodilator administration. Although the sputum weight was higher in the HSP technique, the pellet weight was higher in the P technique with the advantage of less time spent on sputum induction. Furthermore, the samples obtained through the P technique had a higher percentage of viable cells than those obtained through the HSP technique.

During sputum induction or after the end of the technique, there was no respiratory discomfort or limitation in any patient, corroborating the results obtained in adults with asthma (9). Physiotherapy can be offered to patients with benefits of potentially improving the disease condition, quality of life, cardiopulmonary fitness, and maximal inspiratory pressure, as well as reducing symptoms and medication use and improving airway clearance (19). Furthermore, all patients subjected to sputum induction through physiotherapy maneuvers yielded acceptable samples according to the criteria.

The main aim of our study was to demonstrate the efficiency of physiotherapy maneuvers in obtaining sputum samples for cell profile analysis from asthmatic children and adolescents. We observed that sputum induction with physiotherapy maneuvers (P and HSP techniques) was successful in all patients. A similar success was also reported in another study that showed the efficiency of physical or mechanical means of manipulating air flow in mobilizing secretions and producing sputum samples (19,20). In addition, Morsch et al. (9) showed that the inclusion of physiotherapy maneuvers to obtain induced sputum contributed to increasing the final sputum sample weight in asthmatic patients. Further, it is important to emphasize that the sputum induction time with physiotherapy maneuvers was the same for all techniques used in this study. In addition, airway clearance techniques have been demonstrated to increase mucus transport and lung function (20).

The fact that all patients who underwent sputum induction through physiotherapy maneuvers yielded adequate samples according to criteria indicate that these techniques of sputum recovery are both efficient and safe.

Meanwhile, when 3% hypertonic saline was used for sputum induction, we obtained a success rate of approximately 90%. Most likely, this high success rate with HS may be related to the fact that all patients had well-controlled asthma and these rates may be reduced in different clinical conditions. Palomino et al. (21) obtained a success rate of 67% in sputum induction in children with asthma. Other studies involving patients with different asthma severities achieved success rates of 56% (22), 70% (17), 84% (23), and 95% (24).

The amount of eosinophils and neutrophils in mucus is a good indicator of airway inflammation and may represent an important diagnosis and prognostic parameter (17,25). Induced sputum analysis is a safe method for assessing inflammatory markers in various diseases, such as controlled and uncontrolled asthma (26), chronic obstructive pulmonary disease (27), and severe pneumonia in children (28).

With respect to the analysis of inflammatory markers in the three induction techniques, there were no significant differences in the percentages of eosinophils, neutrophils, macrophages, or lymphocytes, as the three techniques had the same inflammatory profiles. Physiotherapy maneuvers not only reduce infectious materials and inflammatory mediators from the airways but also decrease or even prevent host-mediated inflammatory tissue damage. This attenuates the mechanical consequences of obstructive secretions, such as hyperinflation, atelectasis, maldistribution of ventilation, ventilation/perfusion mismatch, and increased work of breathing. Physiotherapy maneuvers offer the most cost-effective therapy as well as a shorter time for procedure execution than other techniques (29).

In our study, the physiotherapy maneuvers used were effective and could be responsible for the improvement in airway sputum expectoration. In addition to inflammatory cell recruitment, the physiotherapy maneuvers were able to recruit disrupted epithelial cells, which are also important in disease pathophysiology (30-32). Although it seemed that the percentage of epithelial cells was increased in the physiotherapy group, no significant differences were observed among the groups. Additionally, it was previously described that <80% squamous cells are acceptable (33) and viable cells must be >50% (34) in the sputum samples.

We are also aware of the limitations of the study. The present study had a small sample size and randomization was applied only at study entry but not in the subsequent inductions. Further, we evaluated only the induction time and not the cost-benefit ratio among the techniques for sputum induction. It is important to compare the cost of acquiring the services of a health professional or a physiotherapist for sample collection, as well as the cost of the materials and equipment relevant for each technique. In addition, it would be important to evaluate the inflammatory markers in the supernatant of sputum samples to demonstrate the agreement among the different techniques.

Induction of sputum is considered a minimally invasive examination regardless of the technique used. Nevertheless, the safety of these techniques should be investigated with the application of questionnaires before and after each induction to evaluate the patients’ respiratory discomfort.

This study confirms that sputum induction through physiotherapy maneuvers is safe in asthmatic children and adolescents, and enables physical therapists to mobilize secretions without causing bronchospasm in patients. With the use of physiotherapy maneuvers, it is possible to induce sputum with the same quality as the gold standard technique (hypertonic saline) with no respiratory discomfort or limitations in asthmatic children and adolescents. Additionally, the cost-benefit ratio of the application of this technique is higher than that of the standard technique owing to the use of less materials and equipment.

## CONCLUSION

The physiotherapy sputum collection technique was effective in obtaining viable cells in mucus samples and yielded the same amount of sputum as the gold standard technique (hypertonic saline). In addition, these physiotherapy maneuvers were both safe and useful for sputum induction in asthmatic children and adolescents with well-controlled asthma.

## AUTHOR CONTRIBUTIONS

Felicio-Júnior EL, Barnabé V, de Almeida FM, de Genaro IS, Rodrigues JC, Tibério IFLC, Martins MA, and Saraiva-Romanholo BM contributed to the study design, data analysis, and writing and preparation of the manuscript. Felicio-Júnior EL, de Almeida FM, Eller MCN, Vergani KP, Avona MD, and Kurdejak A contributed to data generation. All authors contributed to revising the manuscript and have read and approved the final version of the manuscript.

## Figures and Tables

**Table 1:** Pulmonary function.

	HS Median (25-75%)	P Median (25-75%)	HSP Median (25-75%)	*p*-value
FEV_1_ predict (L)	2.32 (1.72-3.18)	2.32 (1.72-3.18)	2.32 (1.72-3.18)	-
FEV_1_ pre BD basal (L)	1.91 (1.51-2.74)	1.85 (1.49-2.78)	1.96 (1.49-2.74)	NS
FEV_1_ % of predict	87.50 (76.12-96.09)	85.20 (75.90-94.67)	91.25 (77.62-95.11)	NS
FEV_1_ post BD (L)	2.01 (1.65-3.02)	1.93 (1.59-2.99)	2.20 (1.55-2.98)	NS
FEV_ 1_ post ind (L)	1.96 (1.53-3.02)	2.11 (1.55-3.10)	2.125 (1.52-2.85)	NS
FEV_1_ decrease (%)	0.00 (0.00-0.92)	0.20 (0.00-9.97)	0.00 (0.00-0.37)	NS
PEF predict (L)	5.49 (4.22-6.98)	5.49 (4.22-6.98)	5.49 (4.22-6.98)	-
PEF pre BD basal (L)	3.90 (3.19-6.21)	3.98 (3.26-5.84)	4.14 (3.24-6.02)	NS
PEF post BD (L)	4.33 (3.64-6.47)	4.29 (3.55-6.51)	4.17 (3.55-6.27)	NS
PEF post ind (L)	4.08 (3.29-6.64)	5.06 (3.61-6.35)	4.67 (3.26-6.30)	NS
PEF decrease (%)	0.40 (0.00-14.60)	0.30 (0.00-12.55)	2.15 (0.00-10.00)	NS
FEV_1_/FVC post BD (L)	0.87 (0.81-0.91)	0.83 (0.79-0.89)	0.89 (0.81-0.91)	NS
FEV_1_/FVC post ind (L)	0.85 (0.81-0.90)	0.86 (0.81-0.89)	0.86 (0.78-0.92)	NS
FEV_1_/FVC decrease (%)	2.20 (0.00-4.62)	0.55 (0.00-2.40)	1.10 (0.00-4.32)	NS

HS: hypertonic saline; P: physiotherapy; HSP: hypertonic saline + physiotherapy; L: liters; %: percentage; FEV_1_ predict: forced expiratory volume in the first second - predict; FEV_1_ pre BD basal: forced expiratory volume in the first second - before bronchodilator administration; FEV_1_ % of predict: percentage of the predicted value according to FEV_1_ pre BD; FEV_1_ post BD: forced expiratory volume in the first second - after bronchodilator administration; FEV_1_ post ind: forced expiratory volume in the first second - after induction (hypertonic saline/physiotherapy); FEV_1_ decrease (%): forced expiratory volume in the first second - percentage of decrease; PEF predict: peak expiratory flow - predict; PEF pre BD basal: peak expiratory flow - before bronchodilator administration; PEF post BD: peak expiratory flow - after bronchodilator administration; PEF post ind: peak expiratory flow - after sputum induction; PEF decrease (%): peak expiratory flow - percentage of decrease; FEV_1_/FVC post BD - FVC expelled in the first second of a forced expiration - after bronchodilator administration; FEV_1_/FVC post ind.: FVC expelled in the first second of a forced expiration - after sputum induction; FEV_1_/FVC decrease (%): FVC expelled in the first second of a forced expiration - percentage of decrease; *p*-value: significance; NS: not significant. Data are expressed as medians and interquartile ranges. There are no statistical differences in the pulmonary function measures among the three techniques.

**Table 2:** Induced sputum characteristics.

	HS Median (25-75%)	P Median (25-75%)	HSP Median (25-75%)	*p*-value
Sputum induction time (min)	14 (7-21)	10 (10-10)	17 (17-17)*	0.001
Sputum weight (g)	0.37 (0.19-0.40)	0.36 (0.24-0.44)	0.41 (0.37-0.46)**	0.020
Pellet weight (mg)	0.005 (0.002-0.013)	0.018 (0.003-0.016)**	0.008 (0.003-0.014)	0.031
Total cells ×10^6^ (cell/mL)	28.00 (16.00-46.25)	20.50 (11.75-43.25)	33.00 (27.00-44.75)	NS
Neutrophils (%)	1.33 (0.00-24.83)	1.00 (0.00-5.91)	2.50 (0.00-17.25)	NS
Eosinophils (%)	1.00 (0.00-5.33)	0.00 (0.00-0.75)	1.00 (0.00-6.33)	NS
Macrophages (%)	34.50 (4.33-64.50)	20.00 (4.58-45.25)	29.00 (14.66-58.00)	NS
Lymphocytes (%)	0.33 (0.00-2.00)	0.00 (0.00-1.00)	0.50 (0.00-0.83)	NS
Epithelial cells (%)	42.71 (6.66-91.33)	72.50 (33.50-94.75)	45.33 (23.16-76.50)	NS
Viable cells (%)	94.55 (84.22-99.55)	85.05 (74.25-94.36)	80.85 (68.25-92.25)**	0.008

HS: hypertonic saline; P: physiotherapy; HSP: hypertonic saline + physiotherapy; min: minutes; mg: milligrams, %: percentage; *p*-value: significance;

* : compared with HS and P;

** : compared with HS; NS: not significant. Data are expressed as medians and interquartile ranges. The time of sputum induction was longer with the HSP technique than with the P technique (*p*=0.001). Sputum weight was greater in the HSP technique than in the other techniques (*p*=0.020). The HSP technique yielded fewer viable cells than the other techniques (*p*=0.008).
