# Erratum for: Randomized trial of physiotherapy and hypertonic saline techniques for sputum induction in asthmatic children and adolescents

**DOI:** 10.6061/clinics/2021/e1512err

**Published:** 2021-08-16

CLINICS (Sao Paulo). 2021;76:e1512err

**Erratum for: doi: https://doi.org/10.6061/clinics/2020/e1512, published in 2020.**

In the article **Randomized trial of physiotherapy and hypertonic saline techniques for sputum induction in asthmatic children and adolescents**

Replace **“Karina Pierantozzi Verganid”** for: **“Karina Pierantozzi Vergani”**

and Page 6 – AUTHOR CONTRIBUTIONS

where it reads **“Verganid KP”** replace for **“Vergani KP”**

